# Effects of Circuit Training on Patients with Knee Osteoarthritis: A Systematic Review and Meta-Analysis

**DOI:** 10.3390/healthcare10102041

**Published:** 2022-10-15

**Authors:** Sameer Badri AL-Mhanna, Mahaneem Mohamed, Norhayati Mohd Noor, Monira I. Aldhahi, Hafeez Abiola Afolabi, Yahkub Babatunde Mutalub, Ahmad Adebayo Irekeola, Kizito Eneye Bello, Wan Syaheedah Wan Ghazali

**Affiliations:** 1 Department of Physiology, School of Medical Sciences, Universiti Sains Malaysia, Kubang Kerian 16150, Kelantan, Malaysia; 2 Department of Family Medicine, School of Medical Sciences, Universiti Sains Malaysia, Kubang Kerian 16150, Kelantan, Malaysia; 3 Department of Rehabilitation Sciences, College of Health and Rehabilitation Sciences, Princess Nourah Bint Abdulrahman University, P.O. Box 84428, Riyadh 11671, Saudi Arabia; 4 Department of General Surgery, School of Medical Sciences, Universiti Sains Malaysia, Kubang Kerian 16150, Kelantan, Malaysia; 5 Department of Clinical Pharmacology, College of Medical Sciences, Abubakar Tafawa Balewa University, Bauchi 740272, Nigeria; 6 Department of Medical Microbiology and Parasitology, School of Medical Sciences, Universiti Sains Malaysia, Kubang Kerian 16150, Kelantan, Malaysia; 7 Microbiology Unit, Department of Biological Sciences, College of Natural and Applied Sciences, Summit University Offa, Offa 4412, Nigeria; 8 Department of Microbiology, Kogi State University (Prince Abubakar Audu University), Anyigba 272102, Nigeria

**Keywords:** circuit training, pain, osteoarthritis, knee

## Abstract

The most prevalent joint disease is osteoarthritis (OA), which affects an estimated 240 million individuals worldwide. Knee osteoarthritis (KOA) is one of the top 10 causes of disability worldwide. The aim of this study is to systematically evaluate the effect of circuit training (CT) on patients with KOA. We searched through PubMed, Scopus, ScienceDirect, Cochrane, and Google Scholar up to 12 February 2022. We used random-effects statistical analysis for continuous variables and reported the results as a standardized mean difference (SMD) with 95 percent confidence intervals (CI). Seven trials involving 346 patients were included. A significant improvement in the intervention group was observed for the parameter, pain level (SMD −0.96, 95% CI −1.77 to −0.14; *p* = 0.02; seven trials, 346 participants; high quality evidence), while no significant improvement was found in physical function (SMD 0.03, 95% CI −0.44–0.50; *p* = 0.89; five trials, 294 participants; high-quality evidence), quality of life (SMD −0.25, 95% CI −1.18–0.68; *p* = 0.60; three trials, 205 participants; high-quality evidence), the activity of daily living (SMD 0.81, 95% CI −0.85–2.48; *p* = 0.34; three trials, 223 participants; high-quality evidence), and knee stiffness (SMD −0.65, 95% CI −1.96–0.66; *p* = 0.33; two trials, 71 participants; high-quality evidence). The findings in this meta-analysis suggest that CT could effectively complement the conventional treatment of KOA, particularly in alleviating pain. However, comprehensive data on the guidelines for the CT approach would be needed to adequately examine the effects of CT on quality of life and biochemical markers in patients with KOA.

## 1. Introduction

Osteoarthritis (OA) is the most common joint disease and the main source of chronic pain and disability [1]. Pain is the predominant symptom of OA [1]. Knee osteoarthritis (KOA) is one of the top ten causes of disability worldwide [2,3,4], accounting for more than 80% of the entire OA disease burden [5]. Clinical OA, as defined by symptoms and physical findings, affects about 27 million US individuals and 8.5 million UK individuals [6,7]. OA is more common as people become older; 13.9% of persons aged 25 and above have clinical OA in at least one joint, whereas 33.6% of those aged 65 and above have OA [8]. 

A major symptom that impedes functional ability is pain, and when it affects the knees, it is considerably more destructive to the well-being of the individual [9]. Psychological factors, such as depression, anxiety, and psychological distress, have been associated with KOA pain [10]. Although psychological variables might likely play a role in exacerbating pain, it is also plausible that pain can lead to a depressed mood. In KOA, pain becomes the primary issue since it is a precursor to other detrimental features of the person’s life. For instance, a sedentary lifestyle contributes to reduced muscular strength and gait pattern alterations, leading to slower walking [10,11]. Furthermore, the presence of muscular weakness and joint proprioception impairment limits everyday activities, resulting in a decrease in postural control and a higher risk of falls [12,13]. Individuals with KOA are likely to have pain, functional limitation, and physical restriction. These significantly affect their activities of daily living, work, leisure, sleep, and social activities. The result is a decrease in their quality of life [14,15]. Quality of life is a broad concept covering all aspects of human life, whereas health-related quality of life focuses on the effects of illness, and specifically, on the impact of treatment on quality of life [16]. Quality of life indicates how a person is living and it indirectly implies the standard of the physical, mental, social, and environmental health status of that person [17]. The benefits of exercise on KOA have been extensively studied in the literature. Exercise protocols are designed to decrease pain, enhance muscular strength and joint stability, as well as improve cardiovascular fitness. All of these contribute to increased functioning [18,19,20]. Exercise, has been demonstrated to strengthen the knee joint as well as lower inflammatory cytokines [21,22]. It has been shown that combining training techniques, including resistive and aerobic exercise is beneficial in dealing with the different symptoms associated with OA [23]. Despite the fact that rehabilitation, especially exercise therapy and strength training, is a frequently recommended critical treatment for OA due to its abundant benefits [24,25], the best strategy for exercise programs remains elusive [24]. 

One strategy to combine the benefits of these techniques is circuit training (CT), which promotes cardiovascular and muscular development. The effect of CT on a variety of chronic diseases, including KOA, may be beneficial as CT involves performing a sequence of exercises repeatedly with no rest or with only short resting intervals [26]. CT is a type of exercise involving resistance and callisthenic exercises to maintain raised heart rate throughout the workout [27,28]. This exercise program consists of sets of several exercises performed in order, each activating different muscle groups. The participant progresses briskly from one exercise to the next, with shorter rest intervals than in traditional strength training, resulting in a substantially reduced overall practice time. CT may be more beneficial than traditional aerobic exercise [29,30]. Due to the short total duration of the exercises, many people may engage in the same training session, which encourages participant retention and adherence [31,32,33,34]. It was reported that lack of time is a typical reason for non-participation in physical exercise programs due to their longer duration and lack of a structural program, particularly among patients with severe pain [31]. Therefore, in this regard, expanding the reasons for exercise prescription in knee OA is a gap that needs to be filled [32]. Even though various studies have shown the effect of CT in improving strength, decreasing pain, and improving functioning, to our knowledge, no study has systematically reviewed the effects of CT in people with KOA [33]. Therefore, this meta-analysis study aimed to investigate the effect of CT in patients with KOA.

## 2. Materials and Methods

### 2.1. Protocol and Registration

The protocol for this study was registered in the international prospective register for systematic reviews (PROSPERO) with the registration number CRD42022325630.

### 2.2. Types of Outcome Measures

#### 2.2.1. Primary Outcomes

1. Pain
2. Quality of life.

#### 2.2.2. Secondary Outcomes

1. Physical function;
2. Activity of daily living;
3. Health-related quality of life;
4. Anxiety;
5. Depression;
6. Stiffness;
7. KOA symptom;
8. High-density lipoprotein;
9. Triglycerides.;
10. HDL;
11. Sport and recreation activities.

### 2.3. Data Sources

According to the Medline method, the search strategy was conducted using keywords and Medical Subject Headings with the boolean operators “OR” and “AND” to find relevant literature. Three independent authors (S.A, B.K, and H.A) conducted an electronic literature search up to 12 February 2022. The keywords used were (“exercise” OR” training”) AND (“circuit*”) AND (“Knee osteoarthritis”) (Supplementary File S1).

### 2.4. Eligibility Criteria 

A search of the literature was carried out to identify experiments that investigated the impact of CT on patients with knee OA. Three researchers (S.A, B.K., and H.A.) used the criteria by PICOS category (population, intervention, comparison, outcome, and study design [34]) to examine the extensive texts of the papers and define the inclusion and exclusion criteria. In the case of disagreements, the judgment of a fourth researcher (A.A.I.) was employed. 

#### 2.4.1. Inclusion Criteria

1. Patients with KOA and with no age limit.
2. Publications with no language limitation and with full text available.
3. CT.
4. Randomized controlled trials and controlled clinical studies.

#### 2.4.2. Exclusion Criteria 

Case reports, review articles, letters, commentaries, short communications, studies without intervention, with no control group, and unclear data. 

### 2.5. Study Selection 

Preferred reporting items for systematic reviews and meta-analyses (PRISMA) guidelines were applied in this study. Three authors, S.A., B.K., and H.A., monitored the selection and exclusion of articles using a linear assessment of titles, abstracts, and full texts (in cases of doubt). The remaining articles were entirely screened using the qualifying criteria before making a final selection. This procedure was employed independently and with the help of a fourth researcher (A.A.I.) if any disagreements or uncertainties existed.

### 2.6. Data Extraction 

After reading the full manuscripts, two authors (S.B.A. and A.A.I.) performed independent sampling and data extraction from eligible studies. The studies that were included contained substantial data that were extracted. The data includes the name of the first author, population, year of publication, gender, age of patients, number of patients, and method (exercise name, duration, intensity, sets, frequency, intervention timing, study duration, and outcome measures).

### 2.7. Assessment of Risk of Bias

The risk of bias was checked based on random sequence generation, allocation concealment, blinding of participants and personnel, blinding of outcome assessors, completeness of outcome data, selectivity of outcome reporting, and other bias, as described in the *Cochrane Handbook for Systematic Reviews of Interventions* [35].

### 2.8. Analysis

#### 2.8.1. Measurement of Treatment Effect

To draw forest plots for trials with categorical outcomes, we used relative risk ratios (RR) and 95% confidence intervals (CI), as well as risk differences (RD), estimates and %CI. We intended to analyze continuous data using mean differences (MD) or standardized mean differences (SMD) and 95% of CI where applicable. 

The heterogeneity of the studies was determined through two steps. First, we screened the demographics, contexts, treatments, and outcomes to determine whether there was any noticeable variability. Second, we used the I^2^ statistic [36] to analyze statistical heterogeneity. We performed a subgroup analysis on the duration of intervention when it was feasible.

#### 2.8.2. Sensitivity Analysis

In the studies that were included, we performed a sensitivity analysis to evaluate how the risk of bias influenced sequence generation and allocation concealment.

### 2.9. Summary of Findings Table

To assess the quality of evidence, we used the GRADEpro technique developed by the Cochrane Collaboration. The GRADEpro system assigns four degrees of quality, the highest being randomized trial evidence. It might be downgraded to moderate, low, or even extremely poor-quality evidence depending on the presence of the following four elements: (i) constraints in study design and implementation; (ii) indirectness of evidence; (iii) unexplained heterogeneity or inconsistency of results; (iv) imprecision of outcomes. The GRADEpro application was used to present the evidence quality for each specific outcome, and the evaluation is being phased in alongside the summary of findings (SoF) table [36].

The SoF table is made up of the following elements:

- Key findings that were summarized (participants, comparative, and baseline data, and results) [37];
- Statistical results that have been condensed;
- A summary of the evidence’s quality, the degree of the effect, and the source of information utilized in the assumed risk.

## 3. Results

### 3.1. Included Studies 

**Figure 1:**
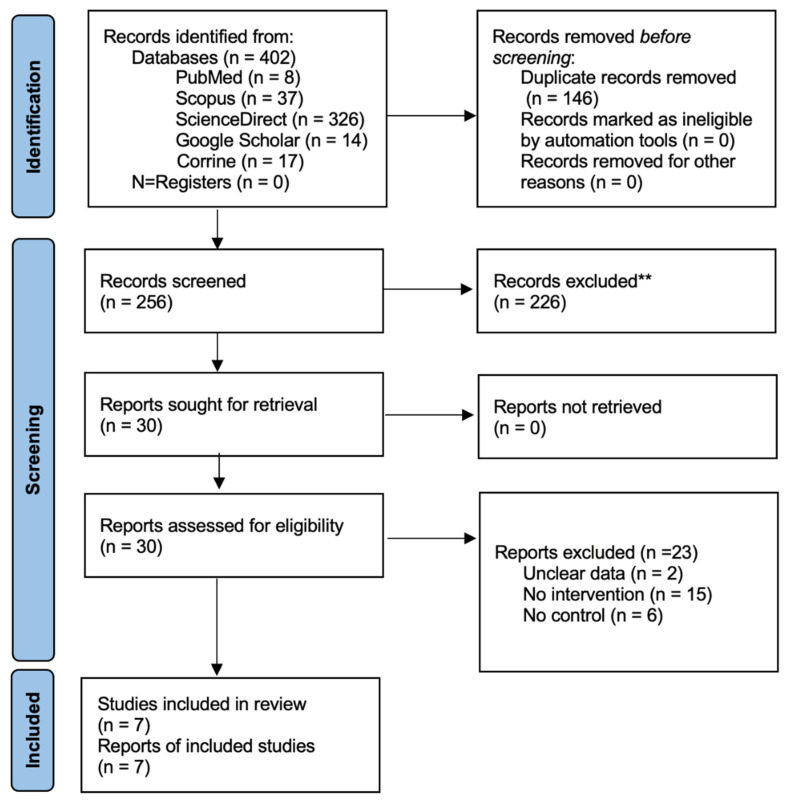
PRISMA flowchart for study selection.

A total of 402 studies were retrieved from the following databases: PubMed, Scopus, Science Direct, Cochrane, and Google scholar as indicated in Figure 1. After identifying duplicate articles, 256 studies were screened for further selection. After reading the titles and abstracts of the articles, a total of 226 were excluded according to pre-set inclusion and exclusion criteria. The full text of the remaining 30 articles was assessed thereafter, of which 23 were excluded. Finally, data were extracted from the seven trials with 346 participants that met the eligibility criteria [33,38,39,40,41,42,43].

### 3.2. Participants Characteristics

Three of the seven trials were from high-income countries [41,42,43], and four were from middle-income countries [33,38,39,40]. Two out of the seven trials recruited their respondents from hospital settings [42,43], while three trials reported enrolling their participants through an informative text or email distributed on social media platforms (Facebook), radio, and newspapers, all of which included interviews with members of the research team [35,43,44]. In one trial, the participants were recruited from the residents of the city of Ribeirao Preto, Sao Paulo, Brazil [39]. Meanwhile, in one trial, information regarding the recruitment of the participants was not provided [38]. Five of the seven trials performed the exercise at healthcare sites [32,39,40,41,42], while one conducted the exercise at both the healthcare site and the participants’ homes [43]. Meanwhile, in one trial, information regarding the exercise site was not stated [38]. In relation to comorbidity, one trial included patients with stable comorbidities such as type 2 diabetes, cardiovascular or respiratory disease, or lower, back, or upper limb pain [42]. Meanwhile, another trial used the Charlson comorbidity index and reported that 45 patients had a single comorbidity, while multi-comorbidities were found in 15 patients [43]. Table 1 describes the characteristics of the included trials.

### 3.3. Intervention Characteristics 

Patients in the included studies were randomly assigned to intervention and control groups. In all seven trials, the intervention was CT [33,38,39,40,41,42,43]. There was a difference in the duration of the intervention among the included studies. In two trials, the intervention was for eight weeks [39,41]. In another trial, the intervention was for one month [38]. In two trials, the intervention was for 14 weeks [33,40]. In one trial, the intervention was for 12 months [42]. In the last trial, the intervention was for 12 weeks [43]. In five trials, the CT was performed at healthcare sites [33,39,40,41,42], while one trial conducted the exercise at the healthcare site and home-based exercise [43]. Meanwhile, one trial did not state information regarding the exercise site [38].

### 3.4. Comparison

In the seven studies, the patients with KOA who underwent CT were compared to the control group who received only standard treatment [33,38,39,40,41,42,43].

### 3.5. Risk of Bias in Included Studies

**Figure 2:**
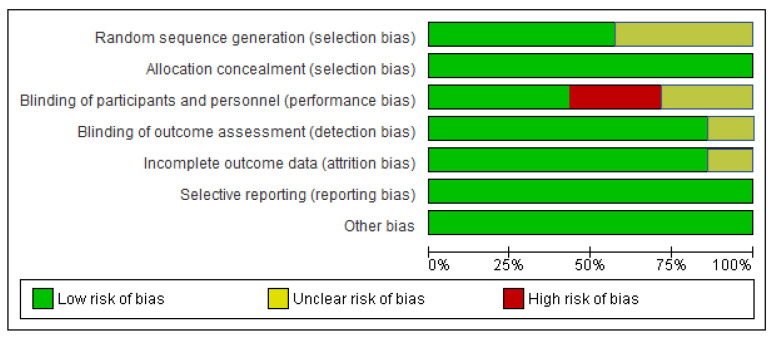
Risk of bias graph: review authors’ judgments about each risk of bias item presented as percentages across all included studies.

**Figure 3:**
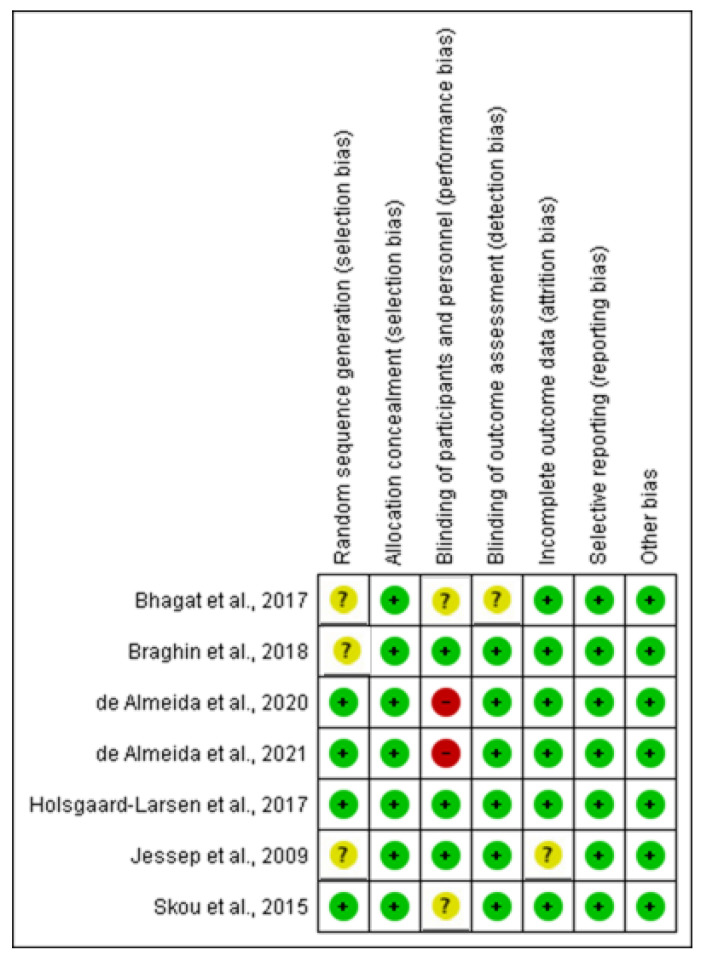
Risk of bias summary: review authors’ judgments about each risk of bias item for each included study [33,38,39,40,41,42,43].

The risk of result assessment bias is depicted in Figure 2 and Figure 3. Figure 2 displays the proportion of studies categorized as low or unclear risk of bias for each risk of bias indicator. Figure 3 depicts the risk of bias indicators for individual studies. The details of the trials are provided in the table of characteristics of included studies.

#### 3.5.1. Random Sequence and Allocation Concealment

In four trials, the randomization method was described, and the random sequence generation was judged to have a low risk of bias [33,40,41,43]. In the remaining three trials, the randomization method was not explained; thus, we judged random sequence generation to have an unclear risk of bias [41,42,45]. Allocation was concealed in two trials by central randomization and was only revealed after baseline assessment [38,42]. In two trials, the allocation was performed by randomization and balance distribution [33,40]. In the remaining three trials, the allocation numbers were concealed in opaque envelopes prepared by a staff member who was independent of the study [42,44,46]. Thus, we judged allocation concealment to have a low risk of bias (Supplementary Materials). 

#### 3.5.2. Blinding of Participants, Personnel, and Outcome Assessment

In three trials, participants were blinded throughout the research procedure, and the trials were deemed to have a low risk of bias [42,44,45]. In two trials, the information regarding the blinding of the participant was not provided and was therefore deemed an unclear risk of bias [38,43]. In two trials, the participants were aware of all exercise procedures and, thus, they were judged to have a high risk of bias [33,40]. The assessors were blinded in six trials and blinding of the outcome assessment was judged to have a low risk of bias [33,39,40,41,42,43]. Meanwhile, one trial did not report if the assessors were blinded, and blinding of the outcome was judged to have an unclear risk of bias [38]. 

#### 3.5.3. Incomplete Outcome Data

Six trials reported that all participants completed the study, and the bias due to incomplete outcome data was judged as low risk [33,38,39,40,41,43]. However, in two trials, a total of four participants, two in each of the intervention and control groups, did not complete the post-treatment assessment because they refused to participate [33,40]. Moreover, in one trial, three participants in the intervention group did not complete the post-treatment assessment. Of the three participants, one died, one canceled, and one was no longer interested. In the control group, six participants did not complete the post-treatment assessment. Of these, one died, two were no longer interested, one canceled, one was unhappy with group allocation, and one had personal or health issues [43]. Meanwhile, the intention to treat analysis was applied. One trial reported that five participants in the intervention group did not complete the post-treatment assessment [42]. One had a hip complication, one had knee surgery, one moved away, and one discontinued the post-treatment assessment. However, the trial did not mention why the fifth participant had not completed the assessment, and the bias due to incomplete outcome data was judged as an unclear risk. Meanwhile, intention to treat analysis was applied and the trial measured the primary outcome at 12 months [42].

#### 3.5.4. Selective Reporting

All seven trials reported the outcomes as specified in their methods section [33,38,39,40,41,42,43] and were regarded as low risk of bias.

#### 3.5.5. Other Potential Sources of Bias

We did not detect any other potential source of bias.

### 3.6. Outcomes

The primary outcomes in this review were pain level and quality of life. Seven trials reported pain levels. Out of the seven, three trials measured the pain level post-intervention using the *Western Ontario and McMaster Universities Osteoarthritis Index* (WOMAC) [35,42,45]. In two trials, the pain level was measured using the Visual Analogue Scale (VAS) [38,40]. Another two trials measured the pain level using the Knee Injury and Osteoarthritis Outcome Score (KOOS) [41,43]. Three trials reported the quality of life using KOOS-quality of life [41,43], and WOMAC-quality of life [38]. The secondary outcomes were physical function, the activities of daily living, health-related quality of life, sport and recreation function, anxiety, depression, stiffness, KOA symptoms, triglycerides, and high-density lipoprotein. Five trials reported on physical function using the WOMAC-physical function [39,42] timed up-and-go test [43], a maximum number of knee bends in 30 s [41], and a 40-m walk test [33]. Three trials reported on the activities of daily living post-intervention using KOOS [41,43] and aggregated functional performance time (AFPT) of four common activities of daily living [42]. Two trials reported on health-related quality of life post-intervention using the EQ-VAS from the EQ-5D 5 Dimensional form 3 level version (EQ-5D-3L) [42,43]. Two trials reported sport and recreation activities post-intervention using KOOS-sport and recreation [41,43]. One trial reported on anxiety post-intervention using the hospital anxiety and depression scale (HADS-A) [42]. One trial reported depression post-intervention using the hospital anxiety and depression scale (HADS-D) [42]. Two trials reported on stiffness post-intervention using WOMAC-stiffness [33,39]. Two trials reported on knee symptoms post-intervention using KOOS [41,43]. One trial reported on triglycerides and high-density lipoprotein using serum samples [33].

#### 3.6.1. Primary Outcomes

The primary outcomes in this review were pain level and quality of life. Seven trials reported pain levels [33,38,39,40,41,42,43], while three trials reported on the quality of life [41,44,46].

##### Knee Pain

**Figure 4:**
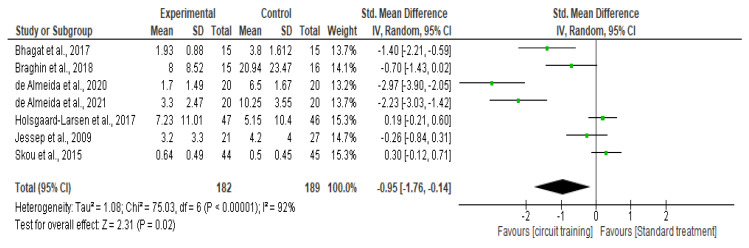
Forest plot analysis of the effect of CT on pain level outcome [33,38,39,40,41,42,43].

There was a significant difference in knee pain outcomes (SMD −0.96, 95% CI −1.77–−0.14; I² statistic = 92%; *p* = 0.02; seven trials, 346 participants; high quality evidence) [33,38,39,40,41,42,43] (Figure 4, Table 2) between the CT group and the standard treatment group.

##### Quality of Life

**Figure 5:**
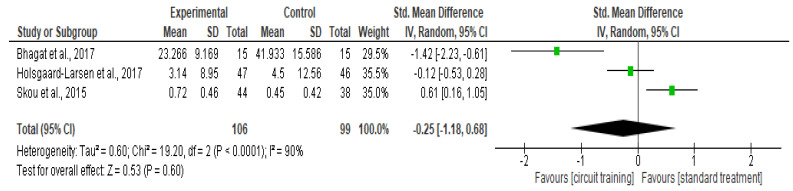
Forest plot analysis of the effect of CT on Quality of life [38,41,43].

There was no difference in the quality-of-life outcomes (SMD −0.25, 95% CI −1.18–0.68; I² statistic = 90%; *p* = 0.60; three trials, 205 participants; high-quality evidence) [38,41,43] (Figure 5, Table 2) between the CT group and standard treatment group.

#### 3.6.2. The Secondary Outcomes

##### Physical Function

**Figure 6:**
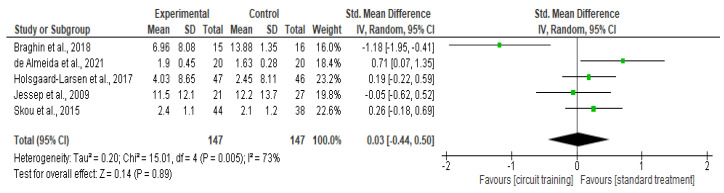
Forest plot analysis of the effect of CT on physical function [33,39,41,42,43].

There was no difference in physical function outcomes (SMD 0.03, 95% CI −0.44–0.50; I² statistic = 73%; *p* = 0.89; five trials, 294 participants; high-quality evidence) [33,39,41,42,43] (Figure 6, Table 2) between the CT group and the standard treatment group.

##### Knee Stiffness 

**Figure 7:**
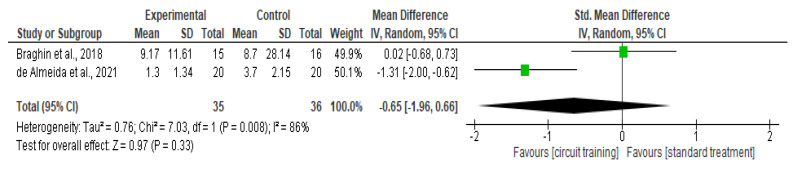
Forest plot analysis of the effect of CT on knee stiffness [33,39].

There was no difference in knee stiffness outcomes (SMD −0.65, 95% CI −1.96–0.66; I² statistic = 86%; *p* = 0.33; two trials, 71 participants; high-quality evidence) [33,39] (Figure 7, Table 2) between the CT group and standard treatment group.

##### Health-Related Quality of Life

**Figure 8:**
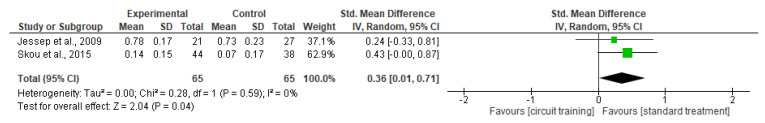
Forest plot analysis of the effect of CT on health-related quality of life [42,43].

There was a significant difference in health-related quality of life outcomes (SMD 0.36, 95% CI −0.01–0.71; I² statistic = 0%; *p* = 0.04; two trials, 130 participants; high-quality evidence) [42,43] (Figure 8, Table 2) between the CT group and standard treatment group. 

##### Knee Symptom

**Figure 9:**
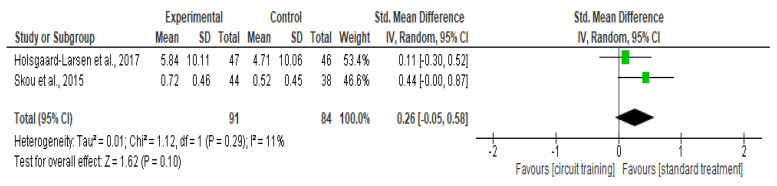
Forest plot analysis of the effect of CT on knee symptom [41,43].

There was no difference in knee symptom outcomes (SMD 0.26, 95% CI −0.05–0.58; I² statistic = 11%; *p* = 0.10; two trials, 175 participants; high-quality evidence) [41,43] (Figure 9, Table 2) between the CT group and standard treatment group.

##### Depression

There was a significant difference in depression outcome (SMD 0.67, 95% CI 0.08–1.26; I² statistic = 0%; *p* = 0.02; one trial, 48 participants; high quality evidence) [42] (Table 2) between the CT group and standard treatment group.

##### Anxiety

There was no difference in anxiety outcome (SMD 0.12, 95% CI −0.45 to −0.69; I² statistic = 0%; *p* = 0.69; one trial, 48 participants; high quality evidence) [42] (Table 2) between the CT group and standard treatment group.

##### Sports Recreation 

**Figure 10:**
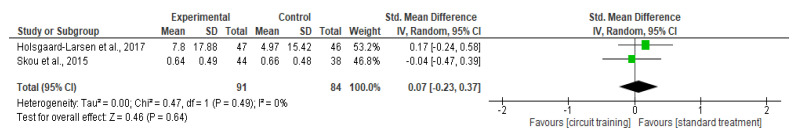
Forest plot analysis of the effect of CT on sports recreation [41,43].

There was no difference in sports recreation outcomes (SMD 0.07, 95% CI −0.23–0.37; I² statistic = 0%; *p* = 0.64; two trials, 175 participants; high-quality evidence) [41,43] (Figure 10, Table 2) between the respiratory rehabilitation group and standard treatment group.

##### The Activity of Daily Living

**Figure 11:**
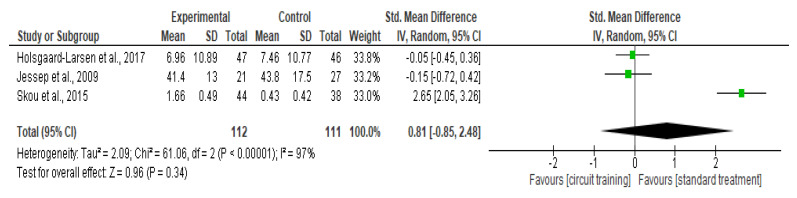
Forest plot analysis of the effect of CT on the activity of daily living [41,42,43].

There was no difference in activity of daily living outcomes (SMD 0.81, 95% CI −0.85–2.48; I² statistic = 97%; *p* = 0.34; three trials, 223 participants; high-quality evidence) [41,42,43] (Figure 11, Table 2) between the CT group and standard treatment group.

##### Triglycerides

There was no difference in triglyceride outcome (SMD 0.01, 95% CI −0.61–0.63; I² statistic = 0%; *p* = 0.98; one trial, 40 participants; high quality evidence) [33] (Table 2) between the CT group and standard treatment group.

##### High-Density Lipoprotein

There was no difference in high density lipoprotein outcome (SMD 0.15, 95% CI −0.47 to −0.77; I² statistic = 0%; *p* = 0.63; one trial, 40 participants; high quality evidence) [33] (Table 2) between the respiratory rehabilitation group and standard treatment group.

## 4. Discussion

### 4.1. Summary of Main Results

The current review was designed to incorporate all randomized controlled trials evaluating the effectiveness of CT among patients with KOA. The activities of daily living, depression, physical function, quality of life, and knee stiffness were not different between the CT and standard treatment groups. There was no difference in the anxiety, sports recreation, HDL, triglyceride, and knee symptom outcomes between the CT and standard treatment groups for the limited number of trials included. Yet, the pain level, depression, and health-related quality of life significantly differed between the CT and standard treatment groups.

### 4.2. Overall Completeness and Applicability of Evidence

We conducted an extensive and elaborate literature review to evaluate the effectiveness of CT among patients with KOA. The RCT included in this review comprehensively illustrate CT outcomes among OA patients. Seven trials were included in the meta-analysis. We found a significant improvement in the intervention group for pain levels.

### 4.3. Quality of the Evidence

The quality of trial evidence ranged from moderate to very low certainty. In many domains, the risk of bias was uncertain or low for most trials. No evidence of selective reporting bias was found. A lack of adequate random sequence generation in the original study and subsequent review may have contributed to treatment effect bias in the original trial and subsequent review. The risk of performance bias was present in two trials. Performance bias was high in two trials because the participants were aware of all the exercise procedures. Two trials reported that four participants (two in each group) did not complete the post-treatment assessment. One trial stated that three participants in the intervention group did not complete the post-treatment assessment. One trial indicated that five participants in the intervention group did not complete the post-treatment assessment. However, in all the aforementioned trials, intention-to-treat analysis was carried out. The study’s random-effects meta-analysis revealed low to moderate heterogeneity. There was no shift in the effect estimate where the random-effects meta-analysis was performed, and although the 95% CI was wider in all cases, the overall quality of evidence contributing to this review, as assessed using the GRADE approach, was moderate to very low.

### 4.4. Potential Biases in the Review Process

We aimed to reduce publication bias by searching different databases without language restrictions and analyzing all relevant papers’ reference lists for extra information. We cannot claim to have identified all the studies in this area with absolute certainty. We were unable to create a funnel plot for publication bias relative to each outcome because there were seven studies included. All included papers satisfied all the inclusion criteria, and we did not introduce any bias through the review process; all studies were thoroughly evaluated, and secondary citations were examined. Though all the studies included in this meta-analysis showed a similar trend, we identified substantial heterogeneity in the knee pain, quality of life, and daily living activities outcomes. Due to limited trials, we were unable to explain this in our analysis.

### 4.5. Agreements and Disagreements with Other Studies or Reviews

To the best of our knowledge, this is the first systematic review and meta-analysis carried out to determine the effectiveness of CT among patients with KOA. Three different reviews examined the effects of exercise on patients with KOA [45,46,47]. Xie, Wang [47] evaluated the effects of a rehabilitation program on patients with KOA and showed significant improvement in pain levels. Meanwhile, there was no significant improvement in physical function among the patients who participated in the rehabilitation program compared with conventional rehabilitation. The study included four trials with a total of 791 patients with KOA. Li, Su [45] included 17 trials with a total of 1705 patients and found that resistance exercise relieves pain, alleviates stiffness, and improves physical function in patients with KOA. Hall, Castelein [46] conducted a systematic review and meta-analysis to evaluate the effects of combined diet and exercise and found moderate improvement in physical function and pain in overweight or obese people with KOA.

## 5. Conclusions

### 5.1. Implications for Practice

In this meta-analysis, CT was found to have a significant effect in improving pain levels in individuals with KOA. Hence, it could effectively complement the conventional treatment of KOA. However, comprehensive data on the guideline for the CT approach would be needed to adequately examine the effects of CT on quality of life and biochemical markers in patients with KOA.

### 5.2. Implications for Research

If further studies are carried out to investigate the application of CT on patients with KOA, they should comprise a detailed pain-assessing test/outcome and outlined safety information. Data on aerobic exercise or combined aerobic and resistance exercise for patients with KOA and other joint problems should also be collated. If studies are carried out in isolated and under-developed regions or settings with no or little access to standard clinical care, the adjuvant treatment should include a comprehensive designed CT program of tolerable duration to improve KOA.

## Figures and Tables

**Table 1:** Characteristics of the included trials.

Reference	Status of the Patient at Intervention	Sample Size	Age/Population	Control	Intervention	Duration	Outcome Measures	Pro Instrument Measure
1. [42]	Grades- Reported comorbidities	48 EX = 21 CO = 27 BMI = -	From 53 to 81 years old UK	Usual care	CT included 10 EX, for two sessions/W for five W. The EX was for 60 (Min). Supervised	12 month	Physical function Pain Anxiety Depression Health-related quality of life Activities of daily living	WOMAC-functioning WOMAC-pain HADS-A HADS-D EQ5D AFPT
2. [38]	Grades I and II OA	30 EX = 15 CO = 15 BMI = -	From 50 to 60 years old India	Usual care	CT includes warm-up for 5 Min, walking, balance training, straight lunges, and one leg balance for 20 min/session, for four W, 3 times/W, 3 and sets.	4 W	QOL Pain	WOMAC-QOL VAS
3. [39]	Grades I, II, and III OA	31 EX = 15 CO = 16 BMI = 30.21 ± 4.63	From 45 to 75 years old Brazil	Usual care	CT includes warm-up for (10 min), strengthening exercises, aerobic exercise on a stationary bicycle (20 min), starting at 65–70% of maximum heart rate (MHR), stretching for (5 min), sitting and standing from a low chair. and walking while changing direction. The exercise was for 60 (min) supervised	8 W	Pain Physical function Stiffness	WOMAC-pain WOMAC- functioning WOMAC
4. [40]	Grades II and III	40 EX = 20 CO = 20 BMI = 26 ± 3.08	From 40 to 65 years old Brazil	Usual care	CT includes a total of 42 exercise sessions and is conducted in three sessions/W. Each session consists of warm-up for 5 (min), CT, and cool-down for (5 min). During the CT, the exercises were classified as light 20 min, moderate 30 min, and intense 40 min. There was a maximum of 30 s of rest between each stage. Supervised	14 W	Pain	VAS
5. [33]	Grades II and III	40 EX = 20 CO = 20 BMI =< 30 kg/m^2^	From 40 to 65 years old Brazil	Usual care	The CT included lower, upper body, and trunk exercises with intensity levels (light, moderate, and intense). The CT for (W 2, 3, and 5) was light exercise moderate exercises were in (W 6, 8, and 9) and intense exercises were in the (W 11, 12, and 14). Between each stage, there was a maximum of 30 s rest. The CT was for 3/W supervised	14 W	Pain Physical function Stiffness HDL Triglycerides	WOMAC-pain 40 M WT WOMAC Serum samples Serum samples
6. [43]	Grades I, II, III, and IV Reported comorbidities	82 EX = 44 CO = 38 BMI = 30.6 ± 5.6	64.8 ± 8.7 years old Denmark	Usual care	CT for lower and upper extremities, consists of warm-up and cooldown periods. CT includes four exercise circles; in between the exercise was postural function: postural orientation, muscle strength, and functional exercises. The intensity was increased if the exercise quality could be maintained. The exercise was two/W with each session lasting 60 min. Supervised	12 W	Pain Knee symptom ADL QOL Physical function General health Sport and recreation	KOOS-Pain KOOS-symptom KOOS-ADL KOOS-QOL Timed Up and Go test EQ-5D 5 KOOS—sport, and recreation
7. [41]	Grades I, II, and III	93 EX = 47 CO = 46 BMI = 27 ± 4 kg/m^2^	From 40 to 70 years old Denmark	Usual care	CT consisted of five-stage: warming up (10 min of aerobic activity, functional exercise, proprioceptive (comprised three exercises), endurance strengthening exercise, and cooling down. The exercise was two/W (each session 60 min) for 8 W. Each exercise included three to four difficulty levels to ensure the progression. Supervised	8 W	Pain Symptom ADL QOL Physical function Sport and recreation	KOOS-Pain KOO—symptom KOOS-ADL KOOS-QOL Maximum number of knee-bendings in 30 s KOOS—sport, and recreation

EX: exercise group; CO: control group; BMI: Body Mass Index; CT: circuit training; HDL: High Density Lipoprotein; W: week; WOMAC: Western Ontario and McMaster Universities Osteoarthritis Index; Min: minute; QOL: quality of life; AFPT: aggregated functional performance time; HADS: hospital anxiety and depression scale; EQ5D: health-related quality of life; KOOS: Knee injury and Osteoarthritis Outcome Score; ADL: activities of daily living; VAS–: Visual Analogue Scale.

**Table 2:** Summary of finding using GRADE quality assessment.

Outcome	Certainty Assessment							No. of Patients		Effect	Certainty
	No. of Studies	Study Design	Risk of Bias	Inconsistency	Indirectness	Imprecision	Other Considerations	Circuit Training	Standard Treatment	Absolute (95% CI)	
Pain level	7	RCT	Serious ^a^	Very serious ^b^	Not serious	Serious ^d^	None	182	182	SMD **0.30 higher** (0.37 higher to 0.56 higher)	⊕◯◯◯ Very low
Quality of life	3	RCT	Not serious	Very serious ^b^	Not serious	Serious ^d^	None	106	99	SMD **0.25 lower** (1.18 lower to 0.68 higher)	⊕◯◯◯ Very low
Physical function	5	RCT	Serious ^a^	Serious ^c^	Not serious	Serious ^d^	None	147	147	SMD **0.03 higher** (0.44 lower to 0.5 higher)	⊕◯◯◯ Very low
Health-related quality of life	2	RCT	Not serious	Not serious	Not serious	Serious ^d^	None	65	65	SMD **0.36 higher** (0.01 higher to 0.71 higher)	⊕⊕⊕◯ Moderate
HDL	1	RCT	Serious ^a^	Serious ^c^	Not serious	Serious ^d^	None	20	20	SMD **0.13 higher** (0.49 lower to 0.75 higher)	⊕◯◯◯ Very low
Triglyceride	1	RCT	Serious ^a^	Serious ^c^	Not serious	Serious ^d^	None	20	20	SMD **0.13 higher** (0.49 lower to 0.75 higher)	⊕◯◯◯ Very low
Depression	1	RCT	Not serious	Serious ^c^	Not serious	Serious ^d^	None	21	27	SMD **0.12 higher** (0.45 lower to 0.69 higher)	⊕⊕◯◯ Low
Anxiety	1	RCT	Not serious	Serious ^c^	Not serious	Serious ^d^	None	21	27	SMD **0.12 higher** (0.45 lower to 0.69 higher)	⊕⊕◯◯ Low
Sports recreation	2	RCT	Not serious	Not serious	Not serious	Serious ^d^	None	91	84	SMD **0.07 higher** (0.23 lower to 0.37 higher)	⊕⊕⊕◯ Moderate
Knee stiffness	2	RCT	Serious ^a^	Serious ^c^	Not serious	Serious ^d^	None	35	36	SMD **0.65 lower** (1.96 lower to 0.66 higher)	⊕◯◯◯ Very low
The activity of daily living	3	RCT	Not serious	Very serious ^b^	Not serious	Serious ^d^	None	112	111	SMD **0.81 higher** (0.85 lower to 2.48 higher)	⊕◯◯◯ Very low
Knee osteoarthritis symptom	2	RCT	Not serious	Not serious	Not serious	Serious ^d^	None	91	84	SMD **0.26 higher** (0.05 lower to 0.58 higher)	⊕⊕⊕◯ Moderate

CI: confidence interval, MD: mean difference, SMD: standardized mean difference, RCT: randomized control trials, ^a^: participants were aware of all exercise procedures, ^b^: there is considerable heterogeneity in the studies, ^c^: there is substantial heterogeneity in the studies, ^d^: the included studies recorded a small sample size for both the control and intervention group.

## Data Availability

Not applicable.

## Supplementary Materials

The following supporting information can be downloaded at: https://www.mdpi.com/article/10.3390/healthcare10102041/s1. Table S1: Risk of bias assessment for studies included in Table 1; File S1: Research question and search strategy employed in the study.
