# Supplementary material for: Effects of Circuit Training on Patients with Knee Osteoarthritis: A Systematic Review and Meta-Analysis
Source: Healthcare (Basel). 2022 Oct 15;10(10):2041. doi: 10.3390/healthcare10102041 (PMC9601599; doi:10.3390/healthcare10102041)
Supplement: Supplementary file 1 [file healthcare-10-02041-s001.zip › healthcare-1868164-supplementary.pdf]

healthcare

MDPI

Supplementary file:

1. Risk of bias assessment for studies included in Table 1

1. Jessep 2009 (Reference [42])

Bias	Authors' Judgement	Support For Judgment
Random sequence generation (selection bias)	Unclear risk	64 eligible patients were consecutively and randomly assigned to the intervention (21) and control group (27)
Allocation concealment (selection bias)	Low risk	"Allocation was concealed by central randomization and only revealed after baseline assessment and held at a center away from Sevenoaks Hospital to ensure concealed allocation"
Blinding of participants and personnel (performance bias) All outcomes	Low risk	"The participants were blinded"
Blinding of outcome assessment (detection bias) All outcomes	Low risk	"The assessor was blinded
Incomplete outcome data (attrition bias) All outcomes	Unclear risk	"Five participants in the intervention group did not complete the post-treatment assessment". "One had a hip complication. One did knee surgery. One moved away. One-stop attending. However, the trial did not mention why the fifth participant had not completed the assessment. Intention to treat analysis was applied.
Selective reporting (reporting bias)	Low risk	Expected outcomes were reported
Other bias	Low risk	Other biases have not been identified

2. Bhagat 2017 (Reference [38])

Bias	Authors' Judgement	Support For Judgement
Random sequence generation (selection bias)	Unclear risk	"The subjects (30) were screened and were put in either of the group A (15)and Group B (15) by simple random sampling with randomized allocation method."
Allocation concealment (selection bias)	Low risk	"Simple random sampling with randomized allocation method was applied".
Blinding of participants and personnel (performance bias) All outcomes	Unclear risk	"Information regarding the blinding of the participant is not provided"
Blinding of outcome assessment (detection bias) All outcomes	Unclear risk	"Information regarding the blinding of the assessor is not provided".
Incomplete outcome data (attrition bias) All outcomes	Low risk	All participants completed the study
Selective reporting (reporting bias)	Low risk	Expected outcomes were reported
Other bias	Low risk	Other biases have not been identified

3. Braghin 2018 (Reference [39])

Bias	Authors' Judgement	Support For Judgement
Random sequence generation (selection bias)	Unclear risk	"A randomized controlled trial". Participants were randomly divided into two groups: Group 1 (n ¼ 15), Participants with symptomatic knee OA and Group 2 (n ¼ 16), Participants with OA symptomatic and no intervention.
Allocation concealment (selection bias)	Low risk	"Group selection was done by a raffle using opaque envelopes from which the volunteers drew, to determine if they were to be in the control or exercise group".
Blinding of participants and personnel (performance bias) All outcomes	Low risk	"The participants were blinded"
Blinding of outcome assessment (detection bias) All outcomes	Low risk	The assessor was blinding
Incomplete outcome data (attrition bias) All outcomes	Low risk	All participants completed the study
Selective reporting (reporting bias)	Low risk	Expected outcomes were reported
Other bias	Low risk	Other biases have not been identified

4. Almeida 2020 (Reference [40])

Bias	Authors' Judgement	Support For Judgement
Random sequence generation (selection bias)	Low risk	"After baseline evaluations, participants were randomized through a randomization website (https://www. random.org/) into three groups: periodized CT, circuit training, ST (strength training), and EP (educational protocol)"
Allocation concealment (selection bias)	Low risk	After baseline evaluations (week 0), participants were randomized through a randomization website (https://www. random.org/) into three groups: periodized CT, circuit training, ST (strength training), and EP (educational protocol)". "A researcher not involved with the evaluations and the allocation was concealed by the randomization and balance distribution".
Blinding of participants and personnel (performance bias) All outcomes	High risk	"Participants were informed about the procedures before participation in the stud." "The physical therapists supervising the exercise intervention sessions were not blinded."
Blinding of outcome assessment (detection bias) All outcomes	Low risk	"The outcome assessor was blinded.
Incomplete outcome data (attrition bias) All outcomes	Low risk	"Two participants in the intervention group did not complete the post-treatment assessment (declined to participate). Another two participants in the control group did not complete the post-treatment assessment (decline to participate)". "Intention to treat analysis was applied".
Selective reporting (reporting bias)	Low risk	Expected outcomes were reported
Other bias	Low risk	Other biases have not been identified

5. Almeida 2021 (Reference [33])

Bias	Authors' Judgement	Support For Judgement
Random sequence generation (selection bias)	Low risk	"After baseline evaluations, participants were randomized through a randomization website (https://www. random.org/) into three groups: periodized CT, (circuit training), ST (strength training), and EP (educational protocol)"
Allocation concealment (selection bias)	Low risk	"After baseline evaluations (week 0), participants were randomized through a randomization website (https://www. random.org/) into three groups: periodized CT, circuit training, ST (strength training), and EP (educational protocol)". "A researcher not involved with the evaluations and allocation was concealed by the randomization and balance distribution".
Blinding of participants and personnel (performance bias) All outcomes	High risk	"Participants were informed about the procedures before participation in the stud." ("Participants were aware of all exercise procedures")"
Blinding of outcome assessment (detection bias) All outcomes	Low risk	The researcher involved with outcome assessments was blinded to group allocation and did not participate in the interventions or any of the testing. The statistician was blind to group allocation before the completion of the statistical analysis.
Incomplete outcome data (attrition bias) All outcomes	Low risk	"Two participants in the intervention group did not complete the post-treatment assessment (declined to participate). Another two participants in the control group did not complete the post-treatment assessment (decline to participate)". "Intention to treat analysis was applied".
Selective reporting (reporting bias)	Low risk	Expected outcomes were reported
Other bias	Low risk	Other biases have not been identified

6. Skou 2015 (Reference [43])

Bias	Authors' Judgement	Support For Judgement
Random sequence generation (selection bias)	Low risk	A randomized controlled trial. "This was a parallel-group assessor-blinded RCT (1:1 treatment allocation) with follow-ups at 3, 6, and 12 months conforming to the CONSORT statement for reporting randomized controlled trial" "A priori, the randomization schedule was generated in permuted blocks of eight, stratified by clinic"
Allocation concealment (selection bias)	Low risk	"The allocation numbers were concealed in opaque envelopes prepared by a staff member independent of the study. The envelopes were accessible to one research assistant at each clinic, only opening them after informed consent and baseline measures had been obtained"
Blinding of participants and personnel (performance bias) All outcomes	Unclear risk	"Information regarding blinding of participants were not provided"

Blinding of outcome assessment (detection bias) All outcomes	Low risk	"The assessors were blinding".
Incomplete outcome data (attrition bias) All outcomes	Low risk	"Three participants in the intervention group did not complete the post-treatment assessment. One is dead. One cancellation or no contact. One no longer interested. Meanwhile, six participants in the control group did not complete the post-treatment assessment. One is dead. Two are no longer interested. One cancellation or no contact. One is unhappy with group allocation. One has personal or health issues. Intention to treat analysis was applied.
Selective reporting (reporting bias)	Low risk	Expected outcomes were reported
Other bias	Low risk	Other biases have not been identified

7. Larsen 2017 (Reference [41])

Bias	Authors' Judgement	Support For Judgement
Random sequence generation (selection bias)	Low risk	"A single-center, randomized (1:1), single-blind, controlled trial, Eligible participants were randomly allocated in permuted blocks of four to six computer-generated a priori by the trial biostatistician (to either exercise or control group"
Allocation concealment (selection bias)	Low risk	"The allocation was concealed in a computer file only accessible by the biostatistician. Individual allocations were held in sealed, opaque, consecutively numbered envelopes, i.e., after the participant had been tested at baseline, the envelope was opened, and the participant was informed about the allocation".
Blinding of participants and personnel (performance bias) All outcomes	Low risk	"The participants were blinded" The assessor was unaware of the study objectives and the randomized distribution of patients to study groups
Blinding of outcome assessment (detection bias) All outcomes	Low risk	"The assessors were blinded"
Incomplete outcome data (attrition bias) All outcomes	Low risk	All participants completed the study
Selective reporting (reporting bias)	Low risk	Expected outcomes were reported
Other bias	Low risk	Other biases have not been identified

2. Research Question

Studies about the effectiveness of circuit training on patients with knee osteoarthritis were selected based on the "PICOS" (PRISMA-P 2016) technique:

"PICOS"

P (population) = knee osteoarthritis patients

I (Intervention) = circuit training

C (Comparison) = Standard treatment

O (Outcome) = physical function and quality of life

S (Study design) = Randomised controlled trial, and controlled clinical studies

3. Search strategy

3.1. PubMed

(("exercise" [Title/Abstract]) OR ("training" [Title/Abstract])) AND ("circuit*" [Title/Abstract])) AND ("Knee osteoarthritis" [Title/Abstract])

3.2. Science direct

("exercise" OR "training") ("circuit") ("Knee osteoarthritis")

3.3. Scopus (37)

TITLE-ABS ("exercise" OR "training") AND TITLE-ABS ("circuit*") AND ("Knee osteoarthritis")

3.4.. Cochrane

("exercise" OR "training") ("circuit") ("Knee osteoarthritis")

3.5. Google Scholar

allintitle("Exercise" OR training) (circuit) (Knee osteoarthritis)
